# Supplementary material for: Pharmacist-Led Deprescribing of Opioids and Benzodiazepines in Older Adults: Examining Implementation and Perceptions
Source: Pharmacy (Basel). 2024 Jul 30;12(4):119. doi: 10.3390/pharmacy12040119 (PMC11360706; doi:10.3390/pharmacy12040119)
Supplement: Supplementary file 1 [file pharmacy-12-00119-s001.zip › pharmacy-2965293-supplementary.pdf]

CDC Qualitative
Semi-structured Interview Guide
Clinic Champions

Thank you for agreeing to participate in today's interview. I would like to provide a few reminders before we begin.

All of our questions are meant to elicit your experiences and perspectives as a clinic participating in the CDC-funded study, Implementation of a Deprescribing Medication Program to Evaluate Falls in Older Adults. This study was developed by researchers from UNC Eshelman School of Pharmacy, UNC School of Medicine, and UNC Center for Aging and Health . In this study, pharmacist consultant recommendations for deprescribing opioids and benzodiazepines occurred from [ ] to [ ]. I will be asking you different questions about this intervention. Your responses will be used to help develop resources for effectively implementing pharmacist-consultant deprescribing programs.

Your participation is voluntary. You can stop or withdraw from the interview at any time or refuse to answer any question. The interview will last approximately 45 minutes.

Before we begin, please confirm that you are the clinic champion of [ ].. To protect confidentiality, the information from the interview will remain anonymous. I will be recording the interview. Is that okay with you? (If response is no, subjects will be excluded)

This is [INTERVIEWER NAME]. I am with interviewee ID [PTT NAME AND ROLE, i.e., "UNC CLINIC #1"]. The current time is [TIME; AM/PM] on [MONTH, DAY, YEAR].

Section 1: Introduction

1. What is your experience deprescribing opioids? Benzodiazepines? **(The characteristics of the individuals who adopt the change)**
   a. *[PROBE] How many years?*
2. Prior to the intervention, what was the culture surrounding deprescribing in your clinic? **(The characteristics of the individuals who adopt the change)**
   a. *PROBE] How often do you and your colleagues talk about deprescribing, if at all?*
   b. *[PROBE] When you talk about deprescribing, what kinds of things do you discuss?*
3. Prior to the intervention,what was the culture surrounding falls? **(The characteristics of the individuals who adopt the change)**
4. What made your clinic decide to participate in the study? **(perception of the innovation)**
   a. *[PROBE] Who in the clinic decided to participate in the study?*
   b. *[PROBE] What role did you play in deciding whether the clinic would participate in the study?*
   c. *[PROBE] What concerns did your clinic have about participating in the study?*
      i. *Do those concerns still exist?*
         1. *If yes, please describe how they continue.*
         2. *If no, please describe how your concerns were resolved.*
      ii. *Do you have any new concerns?*
   d. *[PROBE] What benefits drew your clinic to the study?*
5. Tell me how you became study champion at your clinic? **(The characteristics of the individuals who adopt the change)**

   a. *[PROBE] Have you previously served as a champion for your clinic for other studies, practice innovations, or improvement projects?*

Section 2: Now I'd like to ask you about specific parts of the pharmacist-consultant opioid and benzodiazepine deprescribing study.
<u>Section 2A. This first set of questions will deal with logistics and clinic flow</u>

6. Prior to the intervention, describe your clinic's process for recording patients at high risk for falls? Older adult patients on opioids? Older adult patient on bzds? ***(*****The characteristics of the individuals who adopt the change)**
7. How does the clinic staff use the STEADI question to evaluate patient for falls? **(perception of the innovation)**
8. Did your clinic make any changes in to your patient care process or clinic workflow to accommodate the pharmacist deprescribing recommendations as part of the study?
   a. *[PROBE] Are there any other changes needed that you could think of? (ex: medical assistant staffing, nurse staffing, physician education, nurse education, telemedicine, workflow)*
9. Lack of time has been cited as one of the biggest barriers to beginning the process of deprescribing. To what degree do you believe with this statement?
   a. *[PROBE]* Prior to the intervention, what were some strategies your clinic used to overcome time as a barrier to patient care, if any?
   b. *[PROBE]* How do you feel the pharmacist consultant model has helped with this barrier?
   c. *[PROBE]* How did you fit deprescribing into a patient's appointment prior to the intervention?
   d. *[PROBE]* How did you fit deprescribing into a patient's appointment during to the intervention?
10. In which ways, if any, has the pharmacist-consultant opioid and benzodiazepine deprescribing study impacted your clinic overall? **(perception of the innovation)**
    a. *[PROBE]* Describe the degree of integration of consultant pharmacist into your routine practice at this time, including any facilitators or barriers to integration.
    b. *[PROBE] How did you know whether the new intervention was working at your clinic?*
    a. *[PROBE] What parts of the study worked well at your clinic?*
    b. *PROBE] What deprescribing care changes have been the most difficult to make, and why?*
    c. *[PROBE] If you could change one thing about the study, what would that be and why?*

<u>Section 2B: The next set of questions will deal with staff engagement.</u>

11. Does your clinic currently have a team specifically for fall prevention? deprescribing?
    a. *[PROBE] If yes, How long have you had a team?*
12. Does your clinic currently have a team specifically for deprescribing?
    a. *[PROBE] If yes, How long have you had a team?*
13. As champion, how did you keep clinic staff engaged in the study?
    a. *[PROBE] What barriers, if any, did you experience with keeping staff engaged?*
    b. *[PROBE] Is there any regularly scheduled meeting where deprescribing is routinely discussed? Falls risk? This study?*
       i. *If yes, who attends the meeting and how often is it held?*

   c. *[PROBE] Is there any other way that deprescribing is informally discussed or communicated? Falls risk? This study?*
      ii. *If yes, who initiates that communication and what sorts of people are communicating.*
   d. *[PROBE] Does your clinic use any specific tools or strategies to promote appropriate deprescribing? Fall's assessment? Participation in this study?*

Section 2C: The next set of questions will deal with patient engagement

14. How did the patients respond to the deprescribing recommendations initially?
    a. *[PROBE] How do they respond now?*
    b. *[PROBE] How do you encourage patients to deprescribe opioids and bzds?*
15. What are patient challenges with the deprescribing recommendations, if any?
    a. *[PROBE] What do you do if patients do not want to have their opioid or bzd deprescribed?*

Section 2D: The next set of questions will deal with pharmacist and research team engagement.

16. What are your thoughts about the use of pharmacist in the study? That is, do you think patients received better, worse, or comparable care with regards to deprescribing? **(perception of the innovation)**
    a. *[PROBE] What surprised you the most about the pharmacist consultant model?*
    b. *[PROBE] Do you feel your patients are safer in general when it comes to falls?*
17. As part of this study, clinics were provided resources to assist with deprescribing. These resources included: the Website www.deprescribe.web.unc.edu (deprescribing toolkit), patient education resources, assessments and tools (e.g., Clinical Opiate Withdrawal Scale [COWS], Opioid/Benzodiazepine Taper Agreement), provider resources (e.g., 'Conversations Starters' resource), your practice's clinic champion (i.e., the practice manager or provider who was the point person for our research team). Were there any resources available to you, in addition to those provided by the research team, to assist your clinic in deprescribing?
18. Prior to the intervention, has your clinic provided any fall risk education for staff? Opioid deprescribing? Benzodiazepine deprescribing?
19. To what extent do resource constraints make it difficult to deprescribe opioids and/or benzodiazepines?

Section 3: This next set of questions will be about the impact of COVID and telemedicine.

20. How did the COVID-19 pandemic affect your clinic's participation in the study? **(perception of the innovation)**
    a. *[PROBE] Did your clinic make any changes in* patient care process or clinic workflow *in order to provide the level of care associated with the pharmacist-consultant opioid and benzodiazepine deprescribing study during the pandemic?*
       i. *Are there any other changes needed that you could think of? (ex: medical assistant staffing, nurse staffing, physician education, nurse education, telemedicine)*
       ii. *What deprescribing care changes have been the most difficult to make, and why?*
21. How did tele-medicine play are part in your acceptance of a consultant pharmacist-model?
22. How acceptable is it for your clinic to accept telemedicine as a way to deprescribe opioids? Benzodiazepines? Fall risk?

CDC Qualitative
Semi-structured Interview Guide
Clinic Champions

Section 4: As we near the end of this interview I will now like to get your perspective on the future.

23. What would your clinic need in order to continue a pharmacist-consultant opioid and benzodiazepine deprescribing program?
24. What advice would you give to providers participating in future pharmacist-consultant interventions?
25. 0
